# Anterior Segment Optical Coherence Tomography Analysis of Iris Morphometric Changes Induced by Prostaglandin Analogues Treatment in Patients with Primary Open Angle Glaucoma or Ocular Hypertension

**DOI:** 10.2174/1874364101812010110

**Published:** 2018-06-29

**Authors:** R Mancino, E Di Carlo, D Napoli, A Martucci, A Mauro, RP Sorge, M Cesareo, C Nucci

**Affiliations:** 1 Ophthalmology Unit, Department of Experimental Medicine and Surgery, University of Rome Tor Vergata, Rome, Italy; 2 Department of Ophthalmology, “San Giovanni Evangelista” Hospital, Tivoli (RM), Italy; 3 Laboratory of Biometry, Department of Systems Medicine, University of Rome Tor Vergata, Rome, Italy

**Keywords:** Prostaglandin analogues, Ocular Hypertension, Glaucoma, Visante OCT, Iris thickness, Dilator and sphincter muscle region.

## Abstract

**Background:**

The study aimed to evaluate iris thickness changes in patients with Primary Open Angle Glaucoma (POAG) or Ocular Hypertension (OHT) under treatment with Prostaglandin Analogues (PG).

**Objectives:**

Primary outcome measures were iris thickness at the region of Dilator Muscle Region (DMR) and Sphincter Muscle Region (SMR). DMR/SMR ratio was also evaluated. The secondary outcome was the correlation between PG treatment length and iris parameters.

**Methods:**

The charts of patients with POAG or OHT who underwent Visante OCT were retrospectively selected. The patients were divided in a group using PG for at least 6 months and a group using hypotensive drops not including PG or alpha-adrenergic agonists. A third group included healthy subjects.

**Result:**

98 subjects were selected. Patients with POAG or OHT using PG eyedrops showed a significant iris thickness reduction at DMR compared to healthy subjects and to patients using hypotensive eyedrops not containing PG. Significantly higher SMR thickness values were found in PG group compared to both control groups. DMR/SMR ratio significantly reduced in PG group. No correlation was found between PG treatment length and iris parameters.

**Conclusion:**

The present data indicate that PG treatment induced DMR thickness reduction and an increase in SMR thickness. These changes were not related to the duration of PG exposure.

## 1 INTRODUCTION

Glaucoma is an optic neuropathy characterized by progressive loss of retinal ganglion cells that can conduct to structural optic nerve head changes and visual field alterations [1-3]. Intraocular Pressure (IOP) is the only modifiable risk factor and, until now, represents the only target of antiglaucomatous therapy. The aim of this therapy is to reduce IOP and maintain visual function [4].

A 20-25% reduction of intraocular pressure has been proven to be effective in preventing glaucomatous disease development in the eyes affected by Ocular Hypertension (OHT) and, furthermore, in slowing or halting disease progression in eyes with Primary Open Angle Glaucoma (POAG) [5].

Glaucoma medical therapy is composed of several drugs, both topical eye drops and tablets, with different characteristics of efficacy and tolerability. Five main classes of topical drugs are available: they include prostaglandin analogues and Prostanoids (PG), beta-blockers, topical carbonic anhydrase inhibitors, adrenergic agonists and parasympathomimetics [6].

Prostaglandin analogues are the most effective drugs at lowering IOP and can be suggested as initial medical therapy unless other considerations such as contraindications, cost, side effects, intolerance, or patient refusal preclude this [7].

A recent meta-analysis of randomized clinical trials showed that prostaglandin analogues are effective in reducing intraocular pressure in a range from 31% to 33% compared to maximum IOP measured before starting treatment [8].

The different side effects of prostaglandin analogues include increased lengthening of eyelashes around the eyes and darkening of the iris. This process is not reversible on discontinuation of treatment [9, 10]. The mechanism of iris darkening was largely investigated to exclude the risk to develop cancerous and precancerous lesions induced by cell division [11]. Both clinical [12] and histological [13] studies prove that no proliferative events occurred on iris tissues during PG therapy. Several evidences indicate that the pathophysiological process underlying iris darkening is the increase of melanogenesis within the iris stroma induced by PG treatment [14], but the exact mechanism remains controversial.

The effect of topical prostaglandin analogues on iris structures was investigated *in vivo*, with the use of anterior segment optical coherence tomography, in beagle dogs to evaluate their effects on modifying pupil size and iridocorneal angle anatomy [15].

Anterior segment optical coherence tomography (Visante OCT, Carl Zeiss, Dublin, CA) allows to obtain *in vivo* imaging of the anterior segment structures of the eye [16].

Visante OCT captures and analyzes the cross-sectional images of cornea, anterior chamber, iris and the central portion of the lens using a non-invasive technique based on low coherence interferometry. This tool is particularly useful to visualize narrow angles and to provide accurate measurements of anterior chamber depth [17].

Visante OCT, furthermore, provides fast and high-resolution images, which can be analyzed and measured by calipers.

The aim of the study was to evaluate with the use of anterior segment optical coherence tomography, the occurrence of iris morphometric changes in patients with POAG or OHT under treatment with PG.

## 2 MATERIALS AND METHODS

This pilot, retrospective, case-control, monocentric study adhered to the tenets of Declaration of Helsinki and was approved by the Ethics Committee of Tor Vergata University Hospital, Rome, Italy.

We retrospectively analyzed the charts of patients who underwent fully ophthalmic examination and Visante OCT between 2011 and 2015 at Glaucoma and General Ophthalmology Clinics of the Tor Vergata University Hospital, Rome, Italy.

The inclusion criteria, for each eye of all patients, were: above 18 years of age; diagnosis of POAG or OHT in both eyes according to the criteria of the European Glaucoma Society [18]; intraocular pressure measured by Goldmann applanation tonometry should have been below or equal to 18 mmHg; iridocorneal angle should have been greater or equal to 30° calculated with Visante OCT; previous anterior segment imaging acquired with Visante OCT.

Exclusion criteria were: diagnosis of secondary glaucoma; treatment with alpha-2 adrenergic agonists or other topical non antiglaucomatous eyedrops; previous history of uveitis, rubeoisis iridis, diabetes, tumors and congenital abnormalities of iris, neuro-ophthalmological pathologies, previous episode of acute angle-closure glaucoma, ocular trauma; previous laser or surgical management of glaucoma (iridotomy, trabeculoplasty, trabeculectomy); systemic use of alpha-1-adrenergic receptor antagonists drugs; history of neurological diseases.

Patients fulfilling the inclusion and exclusion criteria were divided into three groups:

1. Patients with the diagnosis of POAG or OHT in both eyes treated with single eye drop containing PG for at least 6 months;
2. Control group formed by patients with diagnosis of POAG or OHT in both eyes treated with ocular hypotensive drops not containing PG for at least 6 months;
3. Control group with healthy subjects, which do not take any kind of eye drops and are not affected by any ocular disease.

The third group was retrospectively selected by the chart of subjects, which spontaneously were gone to the Ophthalmology department in order to perform a screening visit for the prevention of glaucoma. These subjects have undergone a complete examination including the study of the anterior chamber by Visante OCT.

The study analyzed, among all subjects, the data obtained from an accurate ophthalmic examination including general and ophthalmic clinical history, visual acuity, slit-lamp biomicroscopy of the anterior segment, Goldmann applanation tonometry, fundoscopic exam and anterior segment data using Visante OCT.

For all subjects, the anterior segment optical coherence tomography should have been conducted by a single specialist in a room under standardized light conditions (300 lux). Iris cross-sectional images should have been acquired using the “anterior segment single mode” of the Visante OCT. Visante is a Time-Domain OCT characterized by a superluminescent LED with a wavelength of 1,310 nm that allows axial resolution of 18 µm, transverse resolution of 60 µm and a 16 mm x 6 mm scanning field. The anterior segment mode permits to obtain 256 A-scans per line with an acquisition time of 0,125 seconds per single line [19].

To acquire images in a non-accommodative state, the patient’s distance refraction was always used to adjust the fixation target. Visante OCT allows manipulation of the head and image plane to ensure a better visualization of the anterior segment features analyzed. The examiner observed a real-time image of the subject’s eye on the monitor, thus allowing easier manipulation and precision of alignment. Quality control parameters were defined as follows: well-centred image, clearly defined scleral spur and absence of artifacts. Iris crypts were avoided. For each patient, only the cross-sectional images were selected according to the quality control parameters.

The analysis considered only the images with the scanning acquisition plane set at 180° through the centre of the pupil in order to avoid interference with the lid margin. The investigation examined the iris thickness in 2 different positions for all patients: in the Dilator Muscle Region (DMR) and in the Sphincter Muscle Region (SMR), according to the methodology described by Prata *et al*. [20]. Briefly, the study considered the pupillary margin as the medial edge and the temporal periphery of the iris as the temporal landmark.

The region of the dilator muscle was measured at half of the distance between the scleral spur and the pupillary margin and the region of the sphincter muscle at 0,75 mm distance from the pupillary margin Fig. (**1a**-**c**). For the statistical analysis, we considered the mean value of the measurements calculated at two sides of the pupil.

 The ratio between DMR and SMR (DMR/SMR) was also calculated to compensate for possible intersubject variability.

All data analyses were initially listed in an EXCEL database (Microsoft, Redmond, Washington – United States) and the analysis was performed using the Statistical Package for the Social Sciences Windows, version 15.0 (SPSS, Chicago, Illinois, USA). Descriptive statistics consisted of the mean and standard deviation for parameter with Gaussian distributions (after confirmation with histograms and the Kolgomorov-Smironov test). Comparison of continuous variables among groups was performed with ANOVA one-way with Bonferroni correction. Pearson correlation analysis was used to assess the influence of PG treatment and age on iris thickness parameters. A p-value less than 0.05 was considered statistically significant.

## 3 RESULTS

A total of 98 subjects (196 eyes) homogeneous for sex and age were considered for the analysis. 64 patients (128 eyes; 65,30%) were diagnosed with POAG or OHT in both eyes and were divided into two subgroups. The first group included 34 patients (68 eyes; 34,69%) under treatment with PG for at least 6 months Table **1**. 14 of them (41,17%) were in therapy with Latanoprost; 9 with Travoprost (26,47%); 9 used Bimatoprost (26,47%); 2 in therapy with Tafluprost (5,89%). The mean duration of PG treatment was 50,32 ± 32,38 months.

The second group included 30 patients (60 eyes; 30,61%) affected by POAG or OHT treated topically with drugs different from PG or alpha-adrenergic agonists (beta-blockers, topical carbonic anhydrase inhibitors or association) (Table **1**).

The healthy subjects group included 34 subjects (68 eyes; 34,69%) (Table **1**).

Descriptive statistics and iris thickness measurements are shown in (Table **1**).

**Fig. (4):**
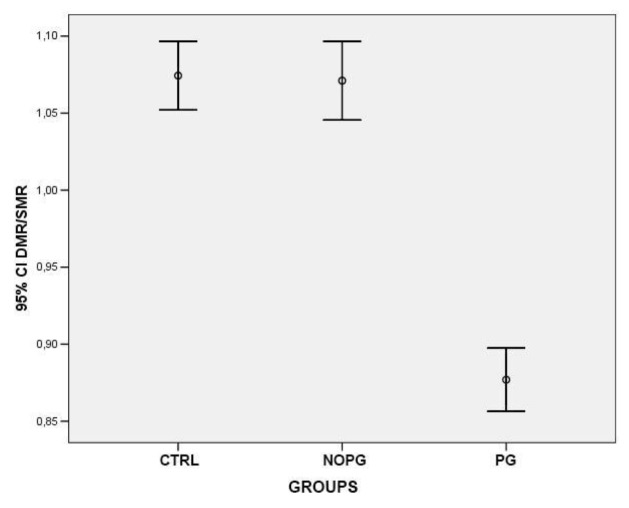
Bonferroni posthoc analysis showed a significant reduction in DMR/SMR ratio in patients with POAG or OHT in therapy with Prostaglandin Analogues (PG) compared to affected patients treated with eyedrops not containing PG or alpha adrenergic agonists (NOPG, p<0,0001) and to control healthy subjects (CTRL, p<0,0001).

At the level of the dilator muscle region, the iris thickness significantly reduced in patients with POAG or OHT under treatment with PG compared to both healthy subjects (0,51 mm±0,067 *vs.* 0,55 mm±0,055; p<0,0001) and patients under treatment with drugs different from PG or alpha-adrenergic agonists (0,51 mm±0,067 *vs.* 0,56 mm±0,057; p<0,0001) Fig. (**2**). On the contrary in the region of the sphincter muscle, the values of iris thickness were significantly higher in patients in therapy with PG compared to both control groups (0,59 mm±0,074 *vs.* 0,52 mm±0,064, healthy controls) (0,59 mm±0,074 *vs.* 0,52 mm±0,049, patients in therapy with drugs different from PG or alpha-adrenergic agonists) (p<0,0001) Fig. (**3**). To confirm the intersubject variability, the ratio between DMR and SMR values (DMR/SMR) was also assessed. Significantly lower values were found in the PG group compared to healthy subjects (0,88±0,085 *vs.* 1,07±0,092; p<0,0001) and to patients in therapy with eyedrops different from PG and alpha-adrenergic agonists (0,88±0,085 *vs.* 1,07±0,099; p < 0,0001) (Fig. **4**).

Comparison of iris thickness values between the group of patients treated with eyedrops not containing PG and healthy subjects did not reveal significant differences (p>0,05) in the DMR region (0,56 mm±0,057 *vs.* 0,55 mm±0,055) and in the SMR region (0,52 mm±0,0049 *vs.* 0,52 mm±0,064). Also the DMR/SMR ratio was not significantly different in these two groups (1,07±0,099 *vs.* 1,07±0,092).

Pearson correlation analysis showed no significant correlation between the duration of prostaglandin analogues eye drops treatment and iris thickness parameters, at the level of DMR (r= 0,007), SMR (r= 0,156) and for DMR/SMR ratio (r= -0,191). Furthermore, there was no significant correlation between age and iris thickness values: DMR (r= -0,154), SMR (r= -0,088) and DMR/SMR ratio (r= -0,047).

## 4 DISCUSSION

The data reports, for the first time, evidence supporting the occurrence of iris morphological alterations, measured with the aim of Visante OCT, in patients affected by primary open-angle glaucoma or ocular hypertension in therapy with prostaglandin analogues.

Visante OCT is a non-invasive method to analyze iris structural changes in treated patients. It allows to measure various anterior chamber parameters such as corneal thickness, anterior chamber depth, anterior chamber angle, and has been used to evaluate corneal scars and ulcers, blebs, ciliary body cleft and iris lesions [21, 22].

In particular, Visante OCT scans revealed a significant reduction in iris thickness at the level of the dilator muscle and a significant increase in the region of the sphincter muscle of patients under treatment with prostaglandin analogues compared to both control groups (healthy subjects and patients under treatment with antiglaucoma drops non containing PG or alpha-adrenergic agonists). These alterations seem to be not influenced by age and not related to the length of treatment. Moreover, the study documented a reduction in DMR/SMR ratio in patients with POAG or OHT using PG compared to the other two groups, strengthening the previous findings that indicate a relevant modification of the iris morphology observed only in the PG group.

Furthermore, no significant differences in iris OCT values were found between eyes with primary open angle glaucoma or ocular hypertension in therapy with hypotensive eye drops not containing prostaglandin analogues or alpha-adrenergic agonists and healthy eyes. Altogether, these data support the hypothesis that the iris changes observed in our study are induced by the use of PG and are not related to glaucoma pathophysiology.

The mechanisms underlying our observations are not known. Many *in vitro* and histopathological studies on PG treatment dealt with the pathogenesis of iris darkening [23-27]. Mainz 1 [28] and Mainz 2 [29] studies assert that short-term therapy with Latanoprost does not produce morphological or cellular proliferation changes in the iris specimens obtained during glaucoma surgery. However, Cracknell *et al*. [30] demonstrated that iris darkening associated with long-term therapy with Latanoprost was due to a small increase in the size of mature melanin granules, especially in the anterior border region.

Moreover, Marquez *et al*. [27] demonstrated that iris specimens obtained from eyes with the previous history of long-term therapy with Latanoprost showed an increased thickness of the anterior limiting layer of iris compared to untreated irises. Therefore, we can hypothesize, with caution, that the increased iris thickness in the Sphincter Muscle Region (SMR) observed in eyes treated with prostaglandin analogues eye drops could be related to this histopathological alteration. However, we should underline that the increased thickness of the anterior limiting layer was homogeneous for the entire iris size and not located in a specific point as we found at the level of the sphincter muscle [27].

Albert *et al*. [31] confirmed previous studies showing increased thickness of the anterior border layer of iris in Latanoprost-treated specimens. Moreover, they found that the number of nuclear invaginations and prominent nucleoli significantly decreased in iris specimens obtained by eyes treated chronically with Latanoprost. These cytological alterations could explain, in part, the significant iris thickness reduction that we found in the region of dilator muscle (DMR) in the eyes in therapy with prostaglandin analogues. However, the results are not clear whether the reduction in nuclear invaginations and prominent nucleoli was concentrated at the level of dilator muscle, thence these data need to be considered with caution.

Few reports focused on the effects *in vivo* of Prostaglandin analogues on the anterior segment structures. Tsai *et al* [15], investigated the prophylactic efficacy of Latanoprost treatment for primary angle closure glaucoma in normal female beagle dogs with the use of anterior segment OCT. They demonstrated a decrease in pupil diameter, crowding of anterior chamber structures and narrowing of iridocorneal angle. In our study, we have not reported cases of narrowing of the anterior chamber angle. However, studies on humans receiving topical latanoprost have shown no effect or mild effect in accommodation and only minimal changes in pupil’s diameter [32, 33]. Further studies are needed to thoroughly investigate the possible relation between prostaglandin analogues eyedrops and the modification of the anterior chamber structures. The altered iris thickness observed in patients using prostaglandin analogues eye drops needs to be further evaluated in order to find a possible relation between iris morphology and pupil dynamics.

Iris is an extremely mobile structure. We may speculate that muscular alteration may be a contributing cause to pupillary response to light alterations previously described in patients with glaucoma [34]. It has been shown that linear ranges for constriction and for dilatation have sharply defined limits in each individual’s eye [35]. Mechanical factors, such as muscular mass reduction, may influence the extent and speed of pupillary movements.

Prata *et al*. [20] demonstrated iris structural alterations associated with intraoperative floppy iris syndrome in patients with prostatic disease in therapy with systemic alpha-1-adrenergic receptor antagonists (alpha-1-ARA) using SL-OCT. They found a significant reduction in iris thickness in the region of Dilator Muscle Region (DMR) in patients using alpha-1-ARA compared to healthy and untreated controls. The research study also showed the same significant changes iris thickness in the region of DMR in eyes treated with prostaglandin analogues eye drops.

Pathogenesis of Intraoperative Floppy Iris Syndrome (IFIS) seems to be related to progressive loss of iris muscle tone caused by atrophy of iris dilator muscle [36, 37].

Based on the data, the eyes treated with prostaglandin analogues could show a “floppy iris” effect during cataract surgery. Assessing patients with Visante preoperatively, may be helpful to establish a more appropriate pre-operative approach. Nevertheless, further studies are required to establish a possible connection between prostaglandin analogues treatment and Floppy Iris Syndrome during phacoemulsification.

To the best of our knowledge, this is the first report to detect iris morphometric changes obtained with Visante OCT in patients with primary open-angle glaucoma or ocular hypertension using prostaglandin analogues eye drops.

Our study has some limitations; it is retrospective in nature and was conducted on relatively small number of subjects in each subgroup. Particularly, the study did not analyze the effects of any single type of prostaglandin analogues eye drops in order to avoid reducing the overall sample size. These limitations make it difficult to establish any evidence and a cause-effect relationship between prostaglandin analogues treatment and morphological changes in iris thickness. We acknowledge that each group of patients was not classified based on the severity of glaucoma. We supposed to overcome these limitations considering a second control group composed of people affected by primary open-angle glaucoma or ocular hypertension in therapy with eye drops not containing prostaglandin analogues eye drops.

Our observations should further be confirmed by prospective studies including larger sample groups to better understand and characterize the effects of prostaglandin analogues in patients with glaucoma.

## CONCLUSION

The present data suggest a possible modification of the iris morphology in patients affected by OH or POAG under treatment with PG eyedrops. Specifically, the use of Visante OCT has revealed reduction in iris thickness at the level of DMR and an increase in iris thickness at SMR in patients using PG.

The study has demonstrated that these changes are not related to PG time exposure.

## Figures and Tables

**Fig. (1a):**
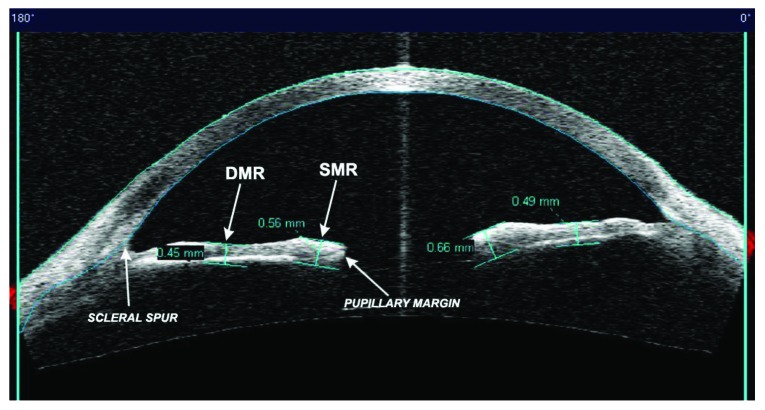
Iris thickness measurements in a patient treated with prostaglandin analogues DMR, dilator muscle region; SMR, sphincter muscle region.

**Fig. (1b):**
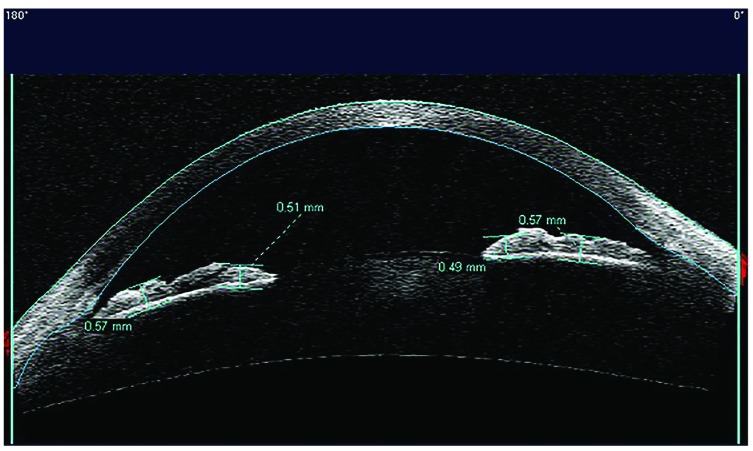
Iris thickness measurements in a patient treated with ocular hypotensive drops not containing PG.

**Fig. (1c):**
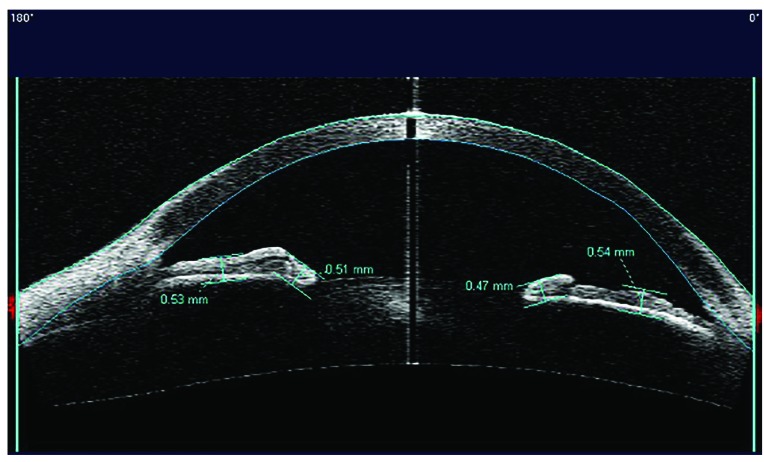
Iris thickness measurements in a healthy subject.

**Fig. (2):**
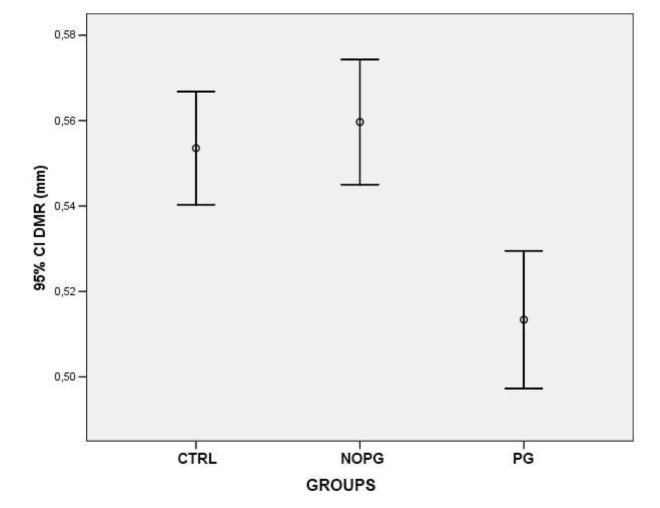
Bonferroni posthoc analysis revealed that topical treatment with Prostaglandin Analogues (PG) significantly reduces iris thickness at the level of the Dilator Muscle Region (DMR) of patients with POAG or OHT as compared to affected patients treated with eyedrops not containing PG or alpha adrenergic agonists (NOPG, p<0,0001) or to control healthy subjects (CTRL, p<0,0001).

**Fig. (3):**
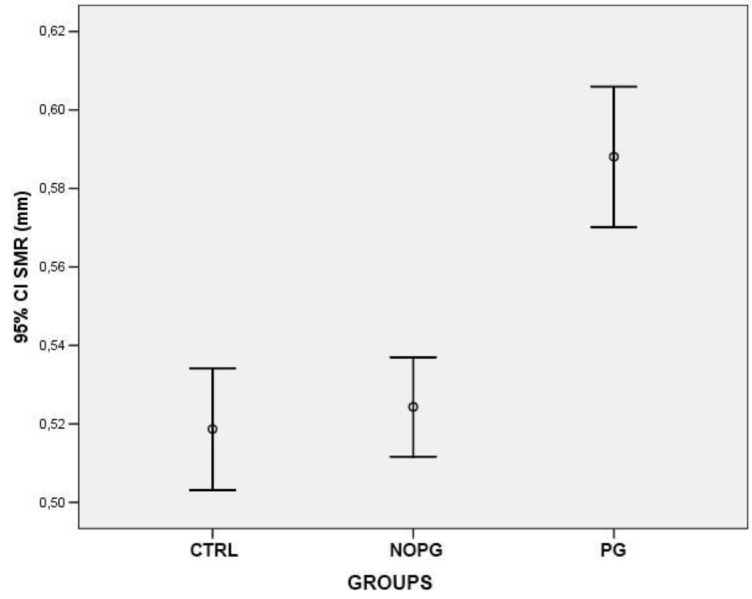
Bonferroni posthoc analysis revealed that topical treatment with Prostaglandin Analogues (PG) significantly increases iris thickness at the level of the Sphincter Muscle Region (SMR) of patients with POAG or OHT as compared to affected patients treated with eyedrops not containing PG or alpha adrenergic agonists (NOPG, p<0,0001) or to control healthy subjects (CTRL, p<0,0001).

**Table 1:** Descriptive statistics and iris thickness values measured in all three groups.

Variable	PG Group	NOPG Group	CTRL Group	ANOVA
	(n=34, 68 eyes)	(n=30, 60 eyes)	(n=34, 68 eyes)	TEST p-value
Age (years)	66,60 ± 14,50	67,10 ± 10,76	64 ± 13,76	0,082
Sex	22 males	17 males	19 males	0,070
	12 females	13 females	15 females	
Iris thickness at DMR	0,51 ± 0,067	0,56 ± 0,057	0,55 ± 0,055	<0,0001
(mm)				
Iris thickness at SMR	0,59 ± 0,074	0,52 ± 0,049	0,52 ± 0,064	<0,0001
(mm)				
DMR/SMR ratio	0,88 ± 0,085	1,07 ± 0,099	1,07 ± 0,092	<0,0001

**DMR**, Dilator Muscle Region; **SMR**, Sphincter Muscle Region.

**CTRL GROUP**, healthy subjects; **PG GROUP**, patients treated with prostaglandin analogues.

**NOPG GROUP**, patients treated with hypotensive eyedrops different from prostaglandin analogues and alpha-adrenergic agonists.
